# Predicting the number of days in court cases using artificial intelligence

**DOI:** 10.1371/journal.pone.0269008

**Published:** 2022-05-26

**Authors:** Raphael Souza de Oliveira, Amilton Sales Reis, Erick Giovani Sperandio Nascimento

**Affiliations:** 1 TRT5 - Regional Labor Court of the 5th Region, Salvador, BA, Brazil; 2 SENAI CIMATEC - Manufacturing and Technology Integrated Campus, Salvador, BA, Brazil; Universiti Malaysia Pahang, MALAYSIA

## Abstract

Brazilian legal system prescribes means of ensuring the prompt processing of court cases, such as the principle of reasonable process duration, the principle of celerity, procedural economy, and due legal process, with a view to optimizing procedural progress. In this context, one of the great challenges of the Brazilian judiciary is to predict the duration of legal cases based on information such as the judge, lawyers, parties involved, subject, monetary values of the case, starting date of the case, etc. Recently, there has been great interest in estimating the duration of various types of events using artificial intelligence algorithms to predict future behaviors based on time series. Thus, this study presents a proof-of-concept for creating and demonstrating a mechanism for predicting the amount of time, after the case is argued in court (time when a case is made available for the magistrate to make the decision), for the magistrate to issue a ruling. Cases from a Regional Labor Court were used as the database, with preparation data in two ways (original and discretization), to test seven machine learning techniques (i) Multilayer Perceptron (MLP); (ii) Gradient Boosting; (iii) Adaboost; (iv) Regressive Stacking; (v) Stacking Regressor with MLP; (vi) Regressive Stacking with Gradient Boosting; and (vii) Support Vector Regression (SVR), and determine which gives the best results. After executing the runs, it was identified that the adaboost technique excelled in the task of estimating the duration for issuing a ruling, as it had the best performance among the tested techniques. Thus, this study shows that it is possible to use machine learning techniques to perform this type of prediction, for the test data set, with an R^2^ of 0.819 and when transformed into levels, an accuracy of 84%.

## Introduction

Human beings have a great ability to recognize patterns [1]. However, they cannot analyze a large dataset in a timely manner, especially when such data does not appear to correlate. One method to aid this recognition process is through statistical/computational methods and data analysis. Nevertheless, some databases have a very large amount of information that, even with the assistance of statistical methods, makes it difficult to perform a deep analysis in search of patterns. An example of this is the databases containing information about legal cases as they have a large amount of data that initially do not directly correlate.

Currently, one of the major challenges in the legal field is to predict the duration of a case based on its information, such as the designated judge, lawyers, parts involved, monetary values, subject matter, etc. The Brazilian legal system prescribes means of ensuring the prompt processing of court cases, such as the principle of reasonable process duration, the principle of celerity, procedural economy, and due legal process, with a view to optimizing procedural progress [2]. Therefore, by having a mechanism for accurately and precisely predicting the duration of a case, it would be possible to anticipate which cases would need to be given greater attention by the court in order to ensure prompt processing while obviously safeguarding the principle of legal certainty. This would be a legal management tool that could have major positive impacts, such as reducing the operational costs for a case, given that the longer the case, the greater the additional costs for allocating the resources needed for its judgment.

Recently, research has shown that machine learning algorithms are powerful tools capable of solving problems that are highly complex or have a high number of nonlinear data, compared to conventional computing methods [3]. Additionally, recent studies have shown that it is possible to precise and accurately estimate the duration of various processes in multiple fields. In this regard, several studies are noteworthy, including [3–13], which used machine learning techniques to estimate the duration of different processes in different fields. Such studies typically estimate the time spent using extensive multidimensional time series datasets [12], which are, in short, a collection of correlated observations made sequentially over time. However, the use of machine learning algorithms is not restricted to time series, as they can also be used in various regression problems.

In this context, a proof-of-concept was developed to assess the possibility of predicting the time for the magistrate to issue the ruling in the Regional Labor Court based on a dataset containing information on more than 300,000 court cases. The prediction should consider the time after the case is argued in court, i.e., when a case is made available for the magistrate to make the decision. Various artificial intelligence (AI) techniques, such as neural networks, regressors, and ensemble techniques, were used comparatively to determine which performed best at achieving the objective of predicting, with good precision and accuracy, the time for issuing a ruling. Their evaluations were made based on the root mean square error (RMSE), normalized root mean square error (NRMSE), factor of two (Fac2) and regression coefficient (R^2^) metrics. Also, this work analyzed how the predictions are explained based on each feature present in the legal processes, using Explainable Artificial Intelligence (XAI) techniques. Thus, this study presents the methodology used in developing the algorithms for predicting the time to issue a ruling, as well as the results achieved by such algorithms. A literature review of recent studies that used AI algorithms in time duration predictions is also presented. Then, the methodology used is described. Finally, the results obtained are discussed, and the conclusions and recommendations for future studies are presented.

## State of the art

There are various AI techniques that seek solutions to problems involving data classification, prediction, or optimization. However, choosing the best technique is not a trivial task, as it requires research, experimentation, and comparison of the models. Many recent studies have shown the feasibility of using AI to predict future behaviors based on time series.

An example is the accurate prediction of the estimated time of arrival (ETA) for commercial flights. If made within an acceptable margin of error, this prediction may result in increased efficiency and airspace capacity [12]. Another example is predicting the average time required for ventilation of patients in intensive care units, which may improve their treatment [11]. These studies show how the analysis of a dataset assists in understanding the behavior of some processes, thereby helping to improve the quality and efficiency of their execution.

As an example, accurate forecasting of the ETA for commercial flights was performed using a system that learned the estimated time based on the history of trajectories, air traffic, airport data, and the weather parameters of the route [12]. This was done using various AI techniques that were trained and tested with data from the 10 busiest flight routes in Spain, with 80% of the data used for training and 20% for testing. The resource-building process generates an extensive dataset of multidimensional time series subjected to time series clustering with dynamic time warping (DTW) to generate a single set of representative resources at each moment in time. Using the root mean square error (RMSE) metric to evaluate the algorithms, the best-performing ones were found to be AdaBoost and Gradient Boosting (GB).

In [4], the use of neural regression algorithms was investigated—specifically multilayer perceptron (MLP) networks with the rectifier linear unit (ReLU) activation function—for estimating the duration of surgery in a dataset possessing 441 characteristics and 86796 tuples, of which 80% were used for training, 8% for validation, and 12% for testing. Comparisons were made between gamma MLP with linear regression and traditional MLP (Gaus sian). The models were evaluated using RMSE, MAE, and negative log-likelihood (NLL), and the best results were found with the MLP Gamma HS and MLP Laplace networks.

In [8], an algorithm based on the extreme learning machine (ELM) technique is proposed to predict the state-of-charge of lithium batteries, in which the voltage and current of the cell are the inputs of the model and the state-of-charge is the output. The ELM used by the authors was a neural network with sigmoid excitation function and 40 hidden layers. The maximum estimation error represents an accuracy of less than 4%.

Another example is given in [13] in which it presents a review of machine learning techniques for forecasting time series energy consumption in residential buildings using current data. The study analyzed the advantages and disadvantages of the three major model categories, namely, (i) unique models; (ii) ensemble models; and (iii) hybrid models.

Recently, there has been some research on the use of AI techniques, more specifically with Natural Language Processing (NLP), in legal cases. However, the researches found, for the most part, use supervised and unsupervised machine learning techniques that do not specifically deal with the target of predicting the number of days of issuing legal cases in courts.

In [14], an approach for classifying articles of law for the Italian legal system based on a learning framework based on Bidirectional Encoder Representations from Transformers (BERT) was presented. Other examples are presented in [15], which performed experiments to detect through the analysis of judgments of the European Court of Human Rights whether the case was judged as a violation of someone’s right or not, and [16] used Glove word vectors generated for the Portuguese Language and Convolutional Neural Network (CNN) to perform the classification of types of documents in judicial proceedings of the Court of Justice of Minas Gerais (TJMG).

In 2021, Sukanya and Priyadarshini [17] carried out a systematic literature review of the challenges faced by the judgment prediction system, which can help lawyers, judges, and civilians predict the rate of profit or loss, time of punishment, and articles from applicable law to new cases, using a deep learning model. The researchers describe in detail the Empirical Literature on Legal Judgment Prediction Methods, the Conceptual Literature on the Text Classification Methods, and transformer model details.

In [18], the researchers sought to detect the degree of similarity between court documents of the Brazilian labor court that can be obtained through unsupervised learning using NLP techniques, namely, (i) term frequency-inverse document frequency (TF-IDF); (ii) Word2Vec with CBoW (Continuous Bag of Words); and (iii) Word2Vec with Skip-gram, both trained for general purposes for the Portuguese language in Brazil.

Despite the variety of studies using process prediction based on time series, to date, no studies that attempted to relate the data on court cases to predict the length of time for issuing a ruling using AI techniques were identified, especially when considering the specificity of the Brazilian legal system. To the best of our knowledge, after a careful literature review, we think this is the first work targeting the prediction of the duration of legal processes in court cases using machine learning.

## Materials and methods

The Python programming language was used in the present study, in which the following supervised machine learning techniques were implemented: (i) neural networks, using the Keras library [19] with TensorFlow [20] as backend; (ii) ensemble methods, using the Sklearn [21] and Mlxtend [22] libraries; and (iii) support vector machine, using the Sklearn and Mlxtend as well.

Some libraries were used for preprocessing, generation of graphs, and calculation of evaluation metrics: (i) Numpy (version 1.15.3), (ii) Pandas (version 0.23.4), (iii) Matplotlib (version 3.0.1), and (iv) Scipy (version 1.1.0). The application development was divided into three steps as follows:

- In the first step, the data were preprocessed such that they could be used by the AI techniques, implementing an initial version of the models using the machine learning techniques chosen.
- In the second step, the chosen techniques were refined to adjust and fine tune the hyperparameters of each algorithm. Finer processing was also performed for adaptation of the data from the cases into a format more suitable for performing the training.
- In the third step, the technique that presented the best performance was evaluated, explaining through the use of the Explainable Artificial Intelligence how the predictions were affected by each characteristic of the lawsuits.

### Dataset

The dataset, which was provided by the Regional Labor Court, contained approximately 8 million records of various cases from this court between 2013 and 2020, totalizing more than 330,000 legal processes. Each record consisted of data about the case identifier, the monetary value of the case, judging organ, subjects, parts involved, type of parts (P: Passive and A: Active), lawyers, magistrate responsible, date of the start of deliberation and date the ruling was issued (Table 1). The ruling date is not presented to the models during the training, and the target is calculated by subtracting the ruling date from the date of the start of deliberation, which is the duration of issuing a ruling on a case. From these features, only the monetary value of the case was represented as a numerical type. The dates, type of part (P: Passive and A: Active) and cases identifier, were represented as date, char, and integer types, respectively. The other features were represented by categorical values using label encoding, generating unique integer values to each class, so that sensitive information is not exposed from the Regional Labor Court, anonymizing them.

**Table 1:** Two cases and their corresponding information in the database. The data were modified to anonymize their content.

Case	XXX-YY-ZZZZ	AAA-BB-CCCC
Monetary value	R$ 91,000.00	R$ 72,349.00
Judging Organ	50	50
Subject	1510	5000
Part	26730	660682
Type of part	P	A
Lawyer	22033	18086
Magistrate	59843	22503
Deliberation start	07/29/2016	08/31/2016
Ruling issued	11/02/2016	12/03/2016

In this database, a single case is represented by various records, as exemplified by Table 2. Thus, out of the nearly 8 million records, there were 333,890 distinct cases, 94 judging organs, 762 subjects, 375,995 parts, two part types, 341,860 lawyers, and 208 magistrates.

**Table 2:** Example of a case with repeated rows contained in the dataset.

Column	Row 1	Row 2
Case	XXX-YY-ZZZZ	XXX-YY-ZZZZ
Monetary value	R$: 38,417.00	R$: 38,417.00
Judging Organ	50	50
Subject	1494	4765
Part	583597	583597
Type of part	A	A
Lawyer	7639	7639
Magistrate	22503	22503
Deliberation start	04/18/2016	04/18/2016
Ruling issued	04/26/2016	04/26/2016

### Preprocessing

Preprocessing is a key step in applying AI techniques. To process and reorganize the data such that they can be used in the AI algorithms (as in Table 2) the data need to be preprocessed. It involves data standardization when there is a large discrepancy between the values presented for the technique, the removal of null values and reorganization and adjustment of the structure of the dataset. It is typically initially necessary for an expert to perform an exploratory analysis of the data being used in order to determine the direction of the preprocessing.

In this study, three forms of preprocessing were used, namely, adjustment of missing values, readjustment of the dataset that was made available, and standardization of the values. These techniques were used first in conjunction with the AI technique execution and then separately.

The adjustment of missing values was the first stage of pre-processing. Many records have lawyers features missing from the available database, because in the lawsuits there may be parts that do not have lawyers. Thus, these missing values were represented with a zero value, indicating, during the learning of the AI techniques, that the part does not have a lawyer.

The second preprocessing step, the readjustment of the dataset, was done in two parts: one for adjustment of the date values and another for adjustment of the table size and organization.

The first part of the readjustment was done in order to make the date format more palatable for the machine learning process. Thus, what had been a chain of characters in the day/month/year format became three separate columns—one for the day, one for the month, and one for the year. This process was performed for only the date of start of deliberation, which resulted in three new attributes in the dataset.

The motivation for the second part of the readjustment of the data was due to wanting to present how different ways to represent the data can impact the learning of the proposed models. In the initial organization of the tables provided, a single case had various rows representing it, in which some column values remained unchanged, while in others, the values changed (Table 2). Initially, the data related to the parts and the lawyer were transformed, according to the type of part, into active or passive part, and active and passive lawyer. Then, two techniques were used, namely, discretization and data close to the crude that will be called the original technique. Next, each of these techniques will be detailed.

- Discretization: In this technique, first the columns whose values changed for the same case identifier were identified. The distinct values of these columns were then grouped by case, and the maximum quantity was sought, taking into consideration the whole table. Finally, for each column identified in the first step, N − 1 new columns were created, where N represents the maximum quantity of distinct values for that column or attribute in the entire dataset, populating these new columns with the original values of each case and 0 for missing values.
- Original: In this technique, the organization of the data was preserved and the case identifier was maintained so that the AI technique could learn that N rows represent the same case.

The third preprocessing step was the standardization or normalization of the values, which is generally necessary when the difference between the minimum and maximum values of the inputs that are part of the dataset is very large. The technique used for the available data was MinMaxScaler, which scales and translates each attribute individually to the interval between 0 and 1. It was thus possible to deliver a consistent dataset for machine learning.

After the preprocessing step, the data were compatible with the inputs expected by the algorithms, and they could be used in the predictive models for training, validation, and testing purposes.

### Implementations of the predictive models

This work involved the development of some supervised machine learning techniques to predict the duration of problems linked to the legal field using regression. Thus, the following neural network-based algorithm was used: multilayer perceptron with backpropagation (MLP).

Among the existing algorithms based on the theory of ensemble methods, algorithms that use the gradient boosting regressor (GBR), adaptive boosting regressor (Adaboost), stacking regressor, stacking regressor with MLP and the stacking regressor with gradient boosting were implemented in this project. Additionally, an algorithm known as support vector regression (SVR) that uses a support vector machine for regression problems was employed.

Table 3 presents the hyperparameters used in implementing the MLP algorithm during the first step of development. Also presented are the hyperparameters used in the second step, which aimed to refine—in order to reduce the evaluation metrics—the hyperparameters used in the first step. Furthermore, both steps used RMSprop as an optimizer and a learning rate of 0.001.

**Table 3:** Hyperparameters used by the MLP model.

Hyperparameter	First Step	Second Step
Hidden Layers	2 with 10 neurons each	3 with 420 neurons each
Activation function	ReLU (rectified linear unit)	Tanh (hyperbolic tangent)
Epochs	100	250
Batch	200	3000

The GBR algorithm was implemented during the first development step. During this step, an initial hyperparameter setting was used, which was changed in the second step, using the GridSearch technique. Table 4 reports the hyper parameters used in both steps for the GBR algorithm.

**Table 4:** Hyperparameters used by the GBR model.

Hyperparameter	First Step	Second Step
n_estimators	10,000	30,000
Max features	sqrt	sqrt
Min samples split	10,000	10,000
Maximum depth	5	5
Learning rate	0.1	0.1

As with the algorithm that used the GBR, two versions of the algorithm that uses the Adaboost technique were implemented—one in the first step and one in the second. Table 5 specifies which hyperparameters are used in each of the steps.

**Table 5:** Hyperparameters used by the adaboost model.

Hyperparameter	First Step	Second Step
base_estimators	Decision Tree Regressor	Decision Tree Regressor with friedman_mse criterion
n_estimators	50	500
Learning rate	0.1	1

The algorithm that used the stacking regressor technique used the StackingCVRegressor method from the Mlxtend library and was developed in conjunction with two regressors (Lasso and Ridge), both from the Scikit-Learn library. This technique was chosen in order to obtain more refinements in the last (third) step of the project. In this step, a specific hyperparameter, the meta regressor, was tested. These tests aimed to find which meta regressor best fit the characteristics of the application in question. For this hyperpa rameter, RandomForest (with n estimators equal to 20 and 40), Bagging (with n estimators equal to 20 and 40) were tested. All final hyperparameter configurations used are described in Table 6.

**Table 6:** Hyperparameters used by the stackingcvregressor model.

Hyperparameter	First Step	Second Step
regressors	Lasso and Ridge	Lasso and Ridge
meta_regressor	RandomForest with 20 n_estimators	RandomForest with 40 n_estimators
use_features_in_secondary	True	True
cv	10	10

Two more ensembles were also made: one combining stacking regressor with MLP and another combining stacking regressor with gradient boosting. For these two experiments, the same hyperparameters as used by the MLP, GB and Stacking Regressor techniques were used, which were detailed in Tables 3, 4 and 6 respectively.

In addition to the algorithms based on neural networks and ensemble methods, an algorithm using SVR was also developed. The settings used by the epsilon hyperparameter were 1.2 and 1.8 in the first and second steps, respectively. In both steps, the kernel parameter was linear, the degree parameter was 3, the gamma parameter was 0.01, and the C parameter was 1000.

Finally, Table 7 presents the final settings for each of the algorithms developed.

**Table 7:** Final settings for the algorithms.

Model	First and Second Steps
MLP	Hidden layers: 3 with 420 neurons each
	Activation function: Tanh
	Epochs: 250
	Batch: 3000
Gradient Boosting	n_estimators: 30,000
	max_features: sqrt
	min_samples_split: 10,000
	Maximum depth: 5
	Learning rate: 0.1
Adaboost	Base estimator: DecisionTreeRegressor with
	friedman_mse criterion
	n_estimators: 500
	Learning rate: 1
Stacking Regressor	regressors: Lasso and Ridge
	meta_regressor: RandomForest with
	40 n_estimators
	use_features_in_secondary: True
	cv: 10
Stacking Regressor with MLP	hyperparameters of the stacking regressor combined with the hyperparameters of the MLP
Stacking Regressor with Gradient Boosting	hyperparameters of the stacking regressor combined with the hyperparameters of the Gradient Boosting
SVR	C: 1000
	Epsilon: 1.8

### Performance and evaluation metrics

The performance evaluation of the prediction algorithms developed for the project was based on the use of the following main metrics related to regression problems:

- Root Mean Square Error (RMSE): Calculates the root mean square difference between the predicted values and the real values.
- Normalized Root Mean Square Error (NRMSE): Calculates the normalized root mean square difference between the predicted values and the real values.
- Regression Coefficient (R^2^): Measure of the fit of a generalized linear statistical model to the observed values. This metric varies between 0 and 1 and indicates, in percentage, how much the model can explain the observed values.
- Factor of 2 (Fac2): Calculates the percentage for the ratio between the predicted and the real values that are between 0.5 and 2, that is, within a factor of 2.

### Model explanation

To evaluate the technique that showed the best performance, in order to explain how the forecasts were affected by each characteristic of the lawsuits, Explainable Artificial Intelligence (XAI) was used. The XAI was proposed by the Defense Advanced Research Projects Agency (DARPA) [23], a division of the American Defense Department that investigates new technologies.

In order to be able to measure the importance of each feature, a framework, called SHAP (SHapley Additive exPlanations), proposed by Lundberg and Lee [24], was used to interpret and understand the predictions. The SHAP framework uses the concept of game theory as a basis for evaluation of coefficients of relative usefulness and regression of the predictors in the model [25].

Thus, the following analyses were carried out: (i) evaluation of the general impact of each feature on the model’s output in relation to the test set; (ii) measuring the contribution of each feature to the increase or decrease of the prediction target; (iii) correlation between future and target through Shapley Value; and analysis of the contribution of each feature to the prediction of each row of a random lawsuit.

## Results and discussions

After the preprocessing (e.g., data standardization and normalization) steps and the construction of the algorithms, the regression algorithms were trained using as input the data separated for training and validation. After the training and validation step, the prediction was made using the test data to evaluate the accuracy and performance of the algorithms. Half of the data were separated for training and validation purposes, and the other half for testing purposes. The portion of the data used for training was presented to the model such that the model could learn the behavior of the cases according to their various attributes, correlating them with the duration of each one. The data used for testing are not presented to the model during the training phase—they are data that have never been viewed by the model and are used to evaluate the model’s real ability to estimate the duration of issuing a ruling on a case, given the case’s attributes. Thus, it is possible to evaluate the accuracy of the model by comparing the predicted duration with the actual duration of each case in the test data portion, quantitatively calculating the error of the model using the statistical metrics presented here.

In this way, using the two preprocessing techniques presented in section “Preprocessing”, each algorithm applied in this study was trained and validated, using the training and validation dataset respectively, with the best hyperparameters found, as shown in Table 7. With the test dataset (with data not known by the models) the prediction was performed reaching the results shown in Table 8.

**Table 8:** Metrics of all algorithms for all preprocessing techniques using test dataset.

Algorithm	Original preprocessing technique	Discretization preprocessing technique
Stacking Regressor	RMSE: 66.552	RMSE: 68.186
	NRMSE: 0.007	NRMSE: 0.008
	R^2^: 0.804	R^2^: 0.785
	Fac2: 0.501	Fac2: 0.439
MLP	RMSE: 79.145	RMSE: 85.351
	NRMSE: 0.009	NRMSE: 0.010
	R^2^: 0.664	R^2^: 0.542
	Fac2: 0.307	Fac2: 0.270
SVR	RMSE: 214.703	RMSE: 221.980
	NRMSE: 0.024	NRMSE: 0.025
	R^2^: —	R^2^: 0.182
	Fac2: 0.059	Fac2: 0.138
**Adaboost**	**RMSE: 65.152**	**RMSE: 66.563**
	**NRMSE: 0.007**	**NRMSE: 0.007**
	**R^2^: 0.819**	**R^2^: 0.804**
	**Fac2: 0.629**	**Fac2: 0.456**
Gradient Boosting	RMSE: 68.734	RMSE: 72.352
	NRMSE: 0.008	NRMSE: 0.008
	R^2^: 0.784	R^2^: 0.741
	Fac2: 0.376	Fac2: 0.348
Stacking Regressor with MLP	RMSE: 71.027	RMSE: 71.733
	NRMSE: 0.008	NRMSE: 0.008
	R^2^: 0.757	R^2^: 0.749
	Fac2: 0.415	Fac2: 0.394
Stacking Regressor with Gradient Boosting	RMSE: 68.092	RMSE: 71.554
	NRMSE: 0.008	NRMSE: 0.008
	R^2^: 0.793	R^2^: 0.753
	Fac2: 0.441	Fac2: 0.387

**Fig 1:**
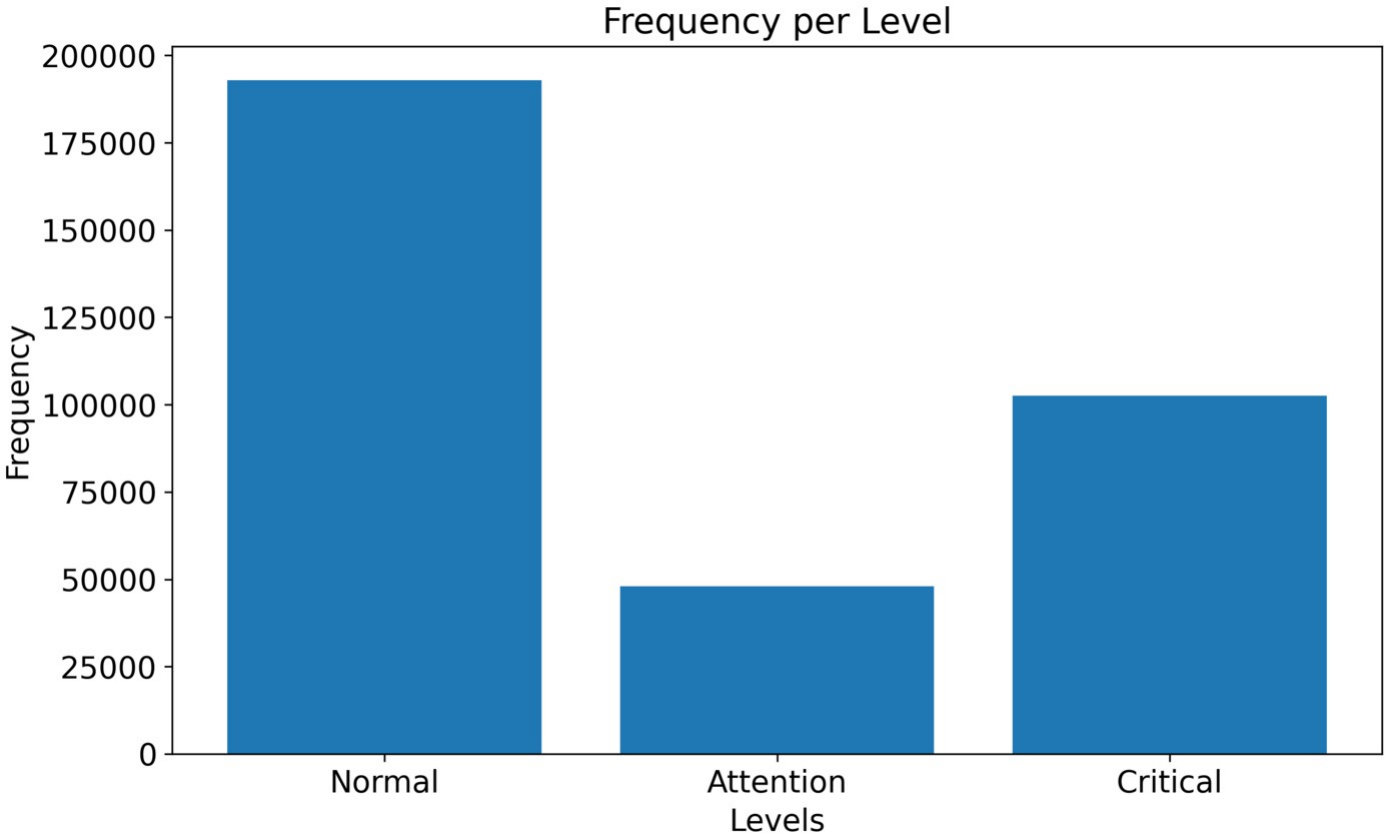
Histogram of the duration of the decision of the processes.

In order to offer an approach to the management of the time of issuing a decision, the processes were grouped into alert levels, which are detailed below, and in addition the frequency histogram was performed according to the three levels grouped (Fig 1):

- Normal Level: set to 0 for the process that obtained the decision time in up to 25 days.
- Attention Level: set to 1 for the process that obtained the decision time between 25 and 60 days.
- Critical Level: set to 2 for the process that obtained the decision time greater than 60 days.

Thus, through the consolidated metrics of the best model (Table 9), it can be seen that the model performed better for processes belonging to the group “Normal Level” and “Critical Level”. This good performance was not verified for the processes belonging to the “group” Attention Level because of the unbalance of the dataset in relation to medium duration processes.

**Table 9:** Consolidated metrics of the model.

Metric	Normal Level	Attention Level	Critical Level	Mean
F1	0.84	0.48	0.75	**0.69**
ROC AUC	0.82	0.72	0.81	**0.78**
PRC AUC	0.80	0.30	0.65	**0.58**
Precision	0.85	0.42	0.79	**0.69**
Recall	0.83	0.56	0.71	**0.70**
Specificity	0.81	0.88	0.92	**0.87**
Accuracy	0.82	0.84	0.85	**0.84**

As highlighted in bold in Table 8, the Adaboost model presented the best result among the models used in this work. Thus, the following XAI analysis will be presented to help correctly interpret the prediction of this model.

**Fig 2:**
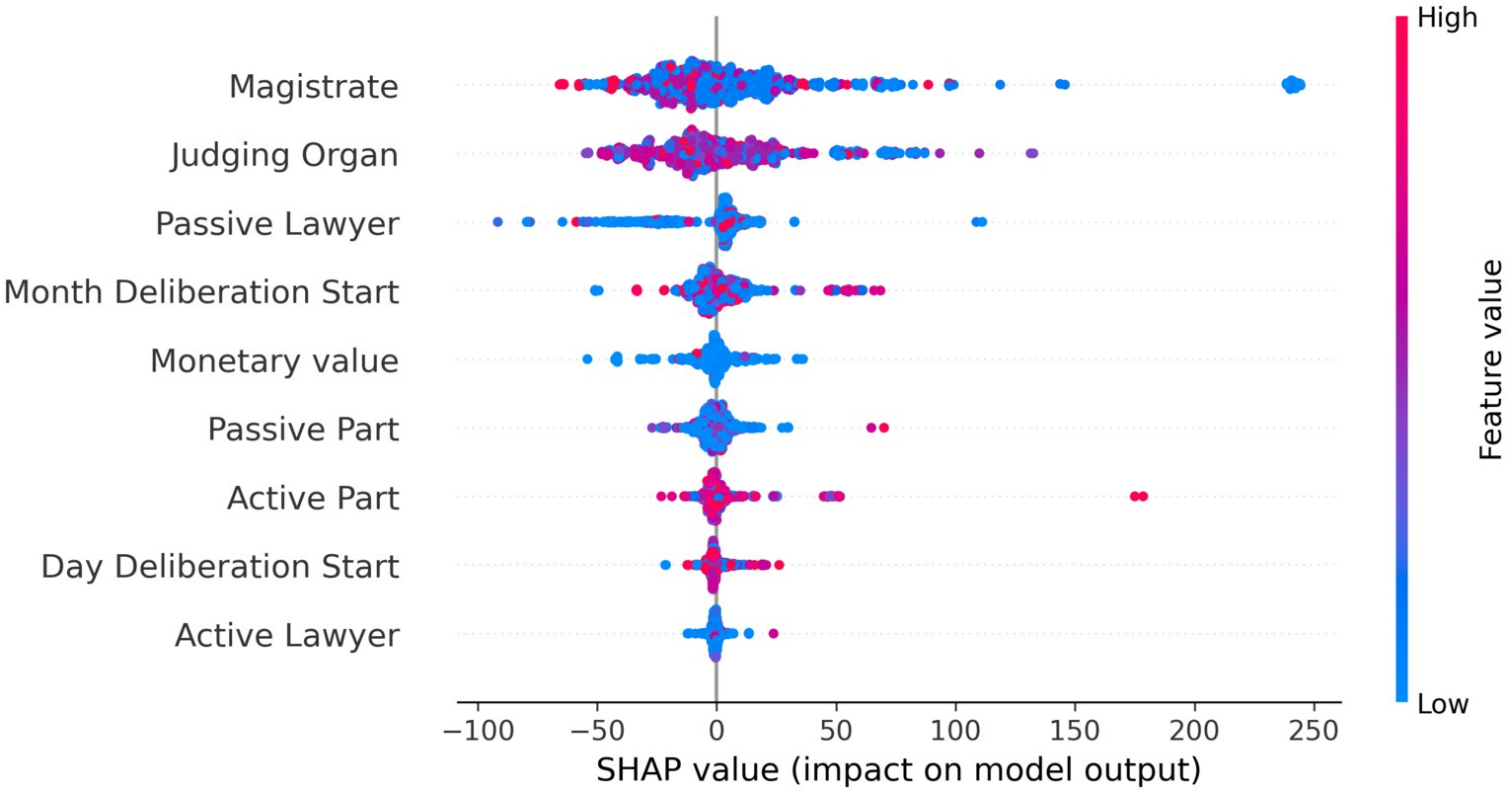
The overall impact of each feature on model output in the test set.

Fig 2 shows the impact on the model output for each feature in order of importance. Thus, it is possible to see that the “magistrate” and the “judging organ” have a greater influence on the target of the prediction (number of judgment days), suggesting that these attributes play an important role in determining the time for issuing rules in legal processes.

**Fig 3:**
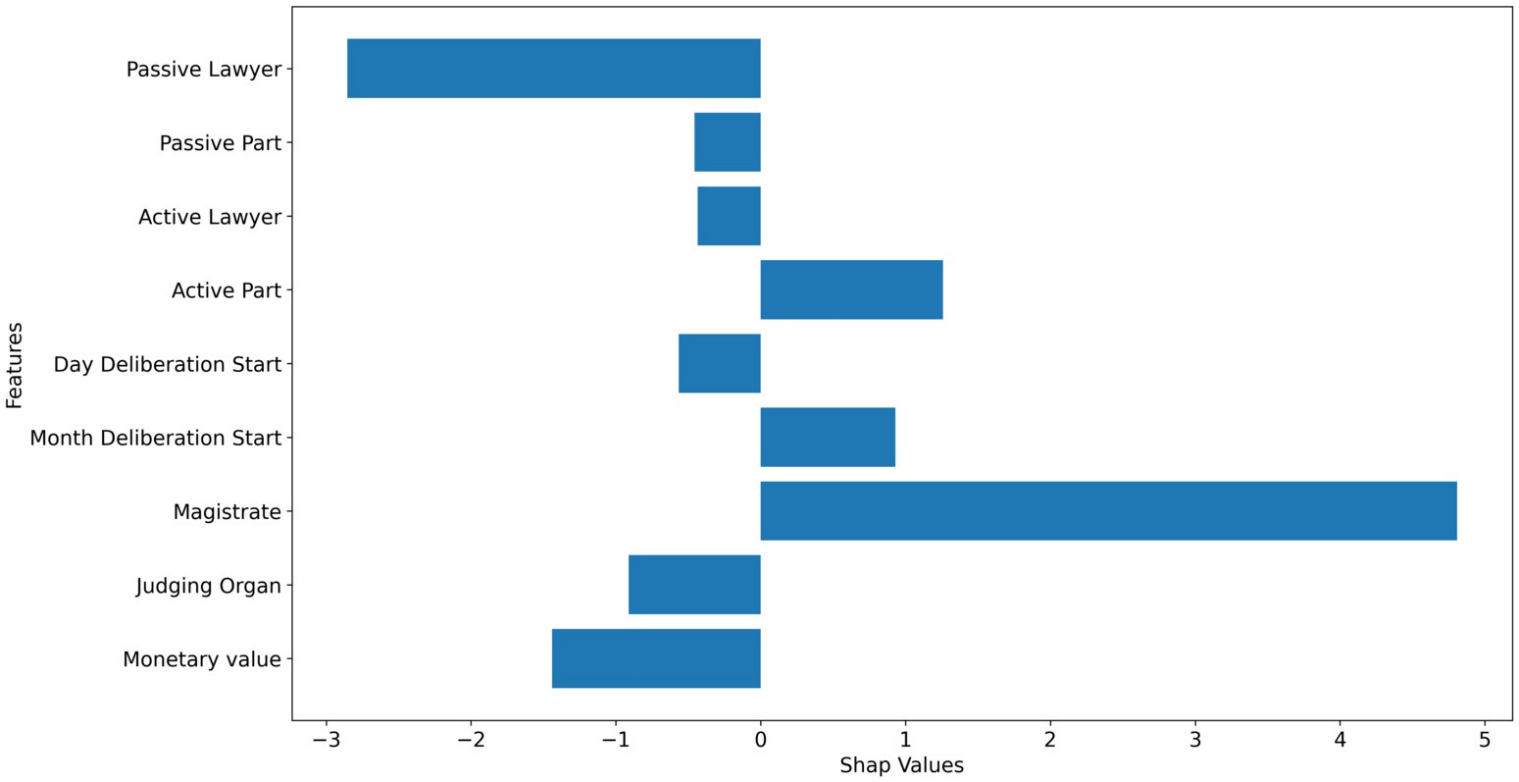
The contribution of each feature for the increase or decrease of the prediction target.

**Fig 4:**
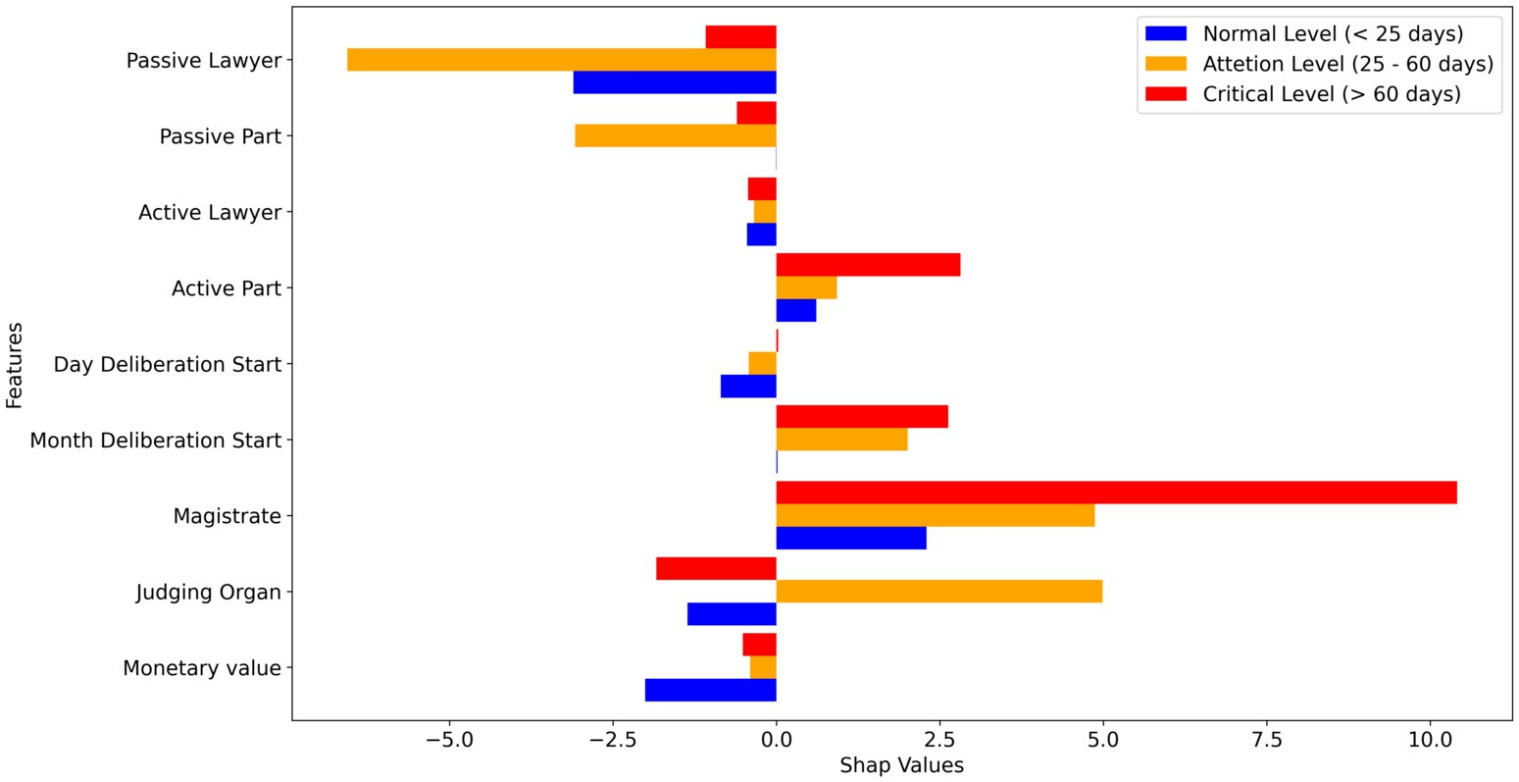
The contribution of each feature for the increase or decrease of the prediction target by alert level.

Figs 3 and 4 highlights how much each feature contributes to increase or decrease the prediction target value for all the test data. Thus, it is possible to infer that in general the features “magistrate” and “active part” drive the forecast upwards (positive SHAP), while “lawyer”, “passive part” and “judging organ” drive the predictions downwards (negative SHAP). It is also noticeable that the month when the deliberation starts plays an interesting role in the predictions, contributing to increasing the duration of the processes. This is consistent with the reality, since there are moments that the court may not have all the work force available due to holidays and vacations periods, which is usually in the Summer season for the legal sector in Brazil.

**Fig 5:**
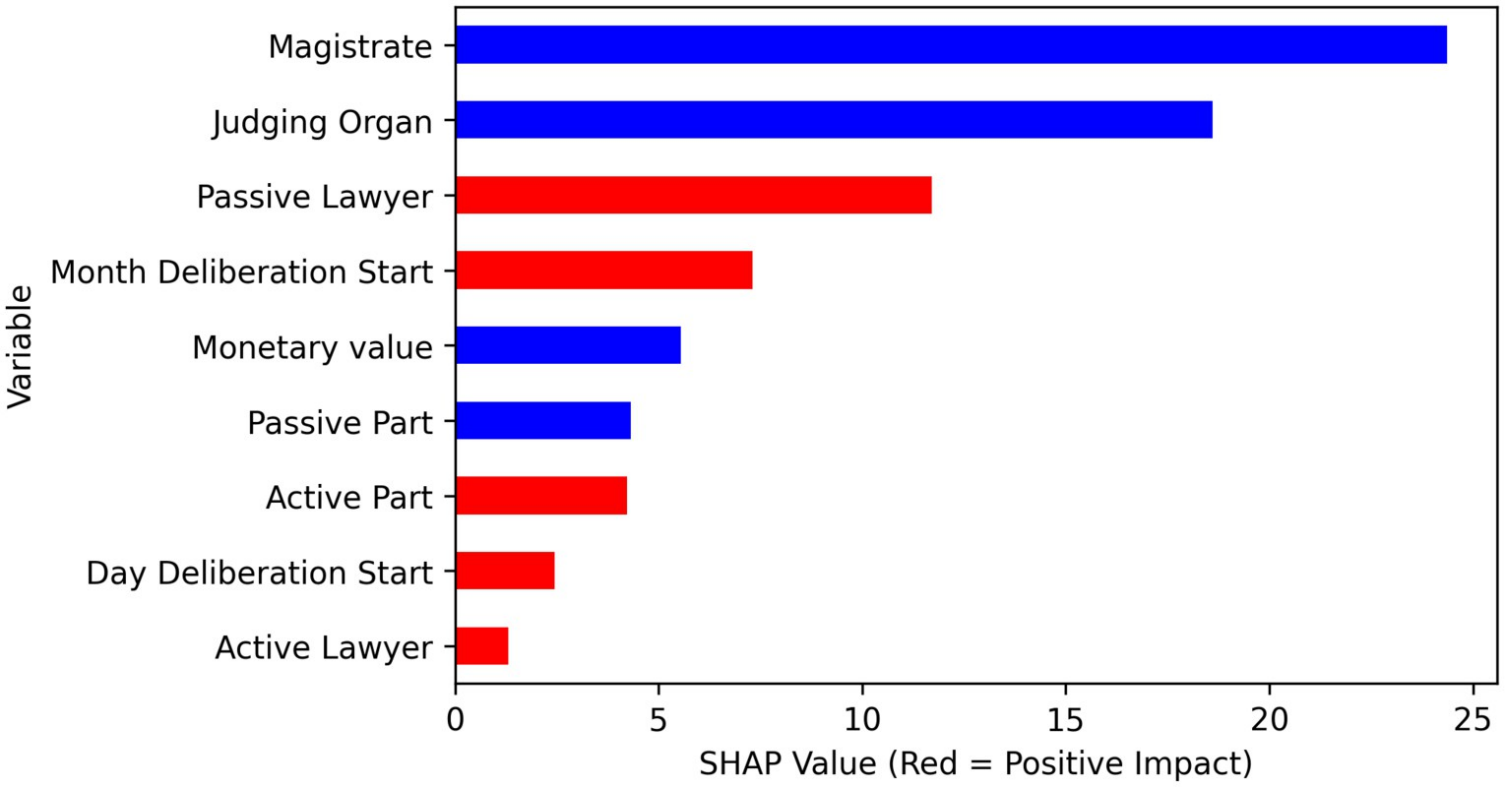
The correlation between the feature and the target of 300 random processes.

Fig 5 shows the correlation between the features and the target of 300 random processes (100 of each level), highlighting with red when it has a positive impact, and with blue when there is a negative impact, highlighting the “magistrate”, “judging organ”, and “passive lawyer” as the most impactful features. These results are consistent with the reality, since they are in most cases the main responsibles for taking decisions or deliberating issues or legal actions in the legal process.

**Fig 6:**
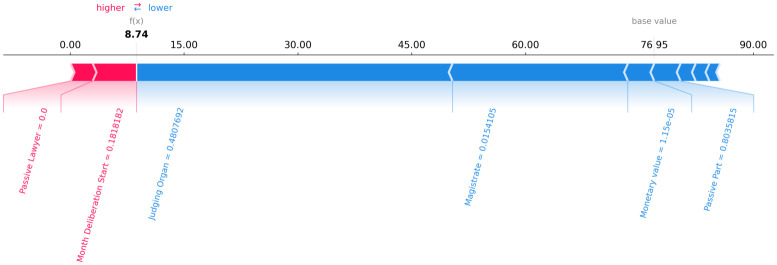
The contribution of each feature to the forecast of each row 1 of a random legal process.

**Fig 7:**
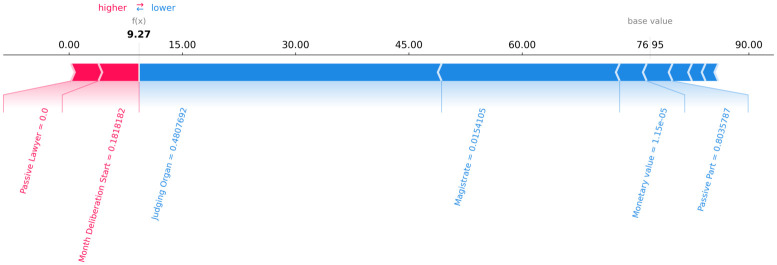
The contribution of each feature to the forecast of each row 2 of a random legal process.

**Fig 8:**
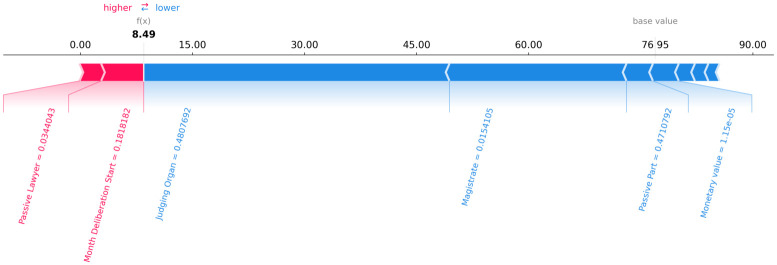
The contribution of each feature to the forecast of each row 3 of a random legal process.

Figs 6–8 present a graphical analysis to illustrate how much each feature contributes to increase or decrease the prediction target value for all rows for a certain random legal process, as an example. From Figs 6–8, given the random legal process example, it is possible to infer that:

- For row 1 (Fig 6): the feature “passive lawyer” drives the forecast upwards, while the “magistrate” and “judging organ” drives the predictions downward.
- For row 2 (Fig 7): the feature “passive lawyer” drives the forecast upwards, while the “magistrate” and “judging organ” drives the predictions downward.
- For row 3 (Fig 8): the feature “passive lawyer” drives the forecast upwards, while the “magistrate” and “judging organ” drive the predictions downward.

**Fig 9:**
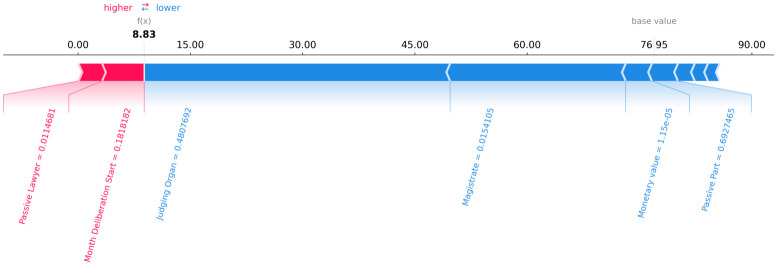
Presents the contribution of each feature to the forecast of the process.

Fig 9 presents how much the feature contributes to increase or decrease the prediction target value for the same random process example, i.e. the average of all rows of the process. Thus, it is possible to infer that the features of “passive lawyer” drive the forecast upwards, while the “magistrate” and “judging organ” drive the predictions downward.

Once the model with the best results has been explained using XAI, as outlined above, it is important to demonstrate that the inference with the trained Adaboost model was done using data never seen before by it during the training as input, which were available in the testing set. The results for the prediction of 15 example cases from the testing set are reported in Table 10.

**Table 10:** Sample of the comparison of inference given by the adaboost algorithm and the real data. The case identifier was modified for anonymization purposes.

Case	Predicted time (days)	Real time (days)
1	269	269
2	8	8
3	1.67	2
4	20	20
5	79	79
6	100.89	100
7	97.25	98
8	2.5	2
9	154	153
10	59	60
11	38.67	38
12	138	139
13	14	13
14	34.33	32
15	4.08	6

It can be observed that the predicted length of time for issuing a ruling was very close to the real observed value, in each case. Considering that the actual duration is an integer value (unlike the predicted duration). The results show how effective the created model is at predicting the length of time for issuing a ruling on a case given the various case attributes.

## Conclusion

The use of AI as a tool for predicting certain characteristics has generally been shown to be a viable and useful solution in scientific and technological fields. In this study, the results were very promising, because the accuracy of the prediction was very high, thus showing that it is possible to predict the length of time for issuing a ruling in court cases by using AI algorithms. Coupled with such algorithms, the data preprocessing step was critically relevant in increasing the prediction quality of the algorithms, significantly improving the results.

Among all evaluated techniques, the Adaboost was found to be the best option for predicting the length of time for issuing a ruling in court cases, given the characteristic of the problem and model on which the algorithm was constructed.

The Adaboost technique best suited the characteristics of the problem, allowing for the development of an inference model that is able to predict, with a low error rate and good performance, the length of time for issuing a ruling in court cases in the Regional Labor Court. However, there was a loss in the quality of the model for the processes belonging to the group “Attention Level”, because the data set used for this study was unbalanced in relation to processes of medium duration. Thus, it is recommended that future studies seek ways to balance the data to the model.

By using the Explainable Artificial Intelligence to explain the predictions, it was possible to verify that the features that most impacted the timing of decisions in the machine learning model are consistent with reality, since, in general, the magistrate, the judging organ and the parties involved (especially the passive part) in the process are determining factors in time for judgment on the case. Thus, it is recommended that future studies seek to compare the results obtained in this research with the results of models using only the most relevant features.

Therefore, from this work, it was possible to advance the current state of the art in the field of machine learning applied to the legal sector, since by predicting the duration of the decision time of the process, it was possible to offer ways for the public administration to adopt practices to accelerate judgment.
